# Artificial microRNA-mediated resistance against Oman strain of tomato yellow leaf curl virus

**DOI:** 10.3389/fpls.2023.1164921

**Published:** 2023-03-30

**Authors:** Maha R. Al-Roshdi, Ume Ammara, Jamal Khan, Abdullah M. Al-Sadi, Muhammad Shafiq Shahid

**Affiliations:** Department of Plant Sciences, College of Agricultural and Marine Sciences, Sultan Qaboos University, Al-Khod, Oman

**Keywords:** RNA interference, agrobacterium-infiltration, artificial microRNA, gene silencing, southern blotting

## Abstract

Tomato yellow leaf curl virus (TYLCV) is a global spreading begomovirus that is exerting a major restraint on global tomato production. In this transgenic approach, an RNA interference (RNAi)-based construct consisting of sequences of an artificial microRNA (amiRNA), a group of small RNA molecules necessary for plant cell development, signal transduction, and stimulus to biotic and abiotic disease was engineered targeting the AC1/Rep gene of the Oman strain of TYLCV-OM. The Rep-amiRNA constructs presented an effective approach in regulating the expression of the Rep gene against TYLCV as a silencing target to create transgenic *Solanum lycopersicum* L. plant tolerance against TYLCV infection. Molecular diagnosis by PCR followed by a Southern hybridization analysis were performed to confirm the effectiveness of agrobacterium-mediated transformation in T0/T1-transformed plants. A substantial decrease in virus replication was observed when T1 transgenic tomato plants were challenged with the TYLCV-OM infectious construct. Although natural resistance options against TYLCV infection are not accessible, the current study proposes that genetically transformed tomato plants expressing amiRNA could be a potential approach for engineering tolerance in plants against TYLCV infection and conceivably for the inhibition of viral diseases against different strains of whitefly-transmitted begomoviruses in Oman.

## 1 Introduction

Tomato (*solanum lycopersicum*) is an important constituent of our daily diet, which has been used for fresh salad, tomato juice, tomato paste, tomato ketchup, and in a variety of other recipes. Therefore, the domestication of the tomato crop has been a major component of the global economy and contributes as a source of subsistence for millions of growers, farmers, and other workers. Similarly to other vegetable crops, tomato also faces several biotic stresses; among them, viral diseases caused by begomoviruses are the most prominent. Tomato yellow leaf curl disease (TYLCD), caused by tomato yellow leaf curl virus (TYLCV), is one of the most devastating begomoviral diseases infecting the tomato crop in diverse tropical, subtropical, and temperate regions around the globe. TYLCV has become the most economically important tomato-invading pathogen, originating from the Middle East and spreading into different countries and regions (Czosnek and Laterrot, 1997; Polston et al., 1999; Font et al., 2000; Moriones and Navas-Castillo, 2000; Lefeuvre et al., 2010; Just et al., 2014; Ramos et al., 2019; Shahid, 2020; AlHudaib et al., 2022). TYLCV is a member of the genus *Begomovirus* of the *Geminiviridae* family, which is distributed globally, and it is difficult to control or eradicate this virus. TYLCV mainly infects tomato crops; however, a wide variety of plant species, ranging from the *Solanaceae* (potato, eggplant, pepper, and tobacco) to *Cucurbitaceae* (pumpkin, watermelon, cucumber, and squash) families, are also severely affected by different strains of this virus. TYLCV is a whitefly-transmitted begomovirus, which causes vein yellowing, severe leaf curling, stunted growth, shortened leaflets, chlorotic and necrotic patches on leaves, and reduced size of the tomato fruit. In many areas, the virus has displayed 100% yield losses depending on the nature of the genotypes, the suitability of the environment, and the development stages of the crop plant.

TYLCV is a circular single-stranded (css) DNA molecule of ∼ 2.7 kb in size, comprising the proteins (C1-C4) required for replication, transcription, suppression of host defense, and (V1-V2) for intracellular and intercellular movement within the host plant (Hanley-Bowdoin et al., 2013). Among the different proteins, the replication initiator protein (C1/Rep) consists of conserved domains for different activities such as the initiation of replication, DNA binding and nicking at A/C in hairpin sequences and ligation, and synthesis of primer and ATPase during DNA replication. Additionally, the Rep protein plays a key role in methylation by regulating MET1: methyltransferase 1 and CMT3: cytosine-5-methyltranserase 3 (Rodríguez-Negrete et al., 2013). In view of the importance of the Rep gene in pathogenicity and its indispensable role in activating plant replication machinery, silencing/suppressing its activity could be an effective approach to limit viral infection/replication (Reyes et al., 2013).

Different strategies have been applied to limit viral transmission, including cultural, chemical, and genetic resistance. In the case of TYLCV, only a limited number of resistance genes were introduced, which restricts the application of breeding for TYLCV resistance, although genetic resistance has been used to develop resistant cultivars for most crops (Kourelis and van der Hoorn, 2018). Hence, there is a dire need for an alternate approach that triggers a single small RNA sequence with limited off-target effects. In engineering genetic resistance against plant viruses, the artificial microRNA (amiRNA) strategy has several advantages over gene silencing based on viral genomic sequences. Therefore, 21 nt amiRNAs with high specificity can be designed to avoid off-target effects. The amiRNA size (nucleotides) is much shorter than virus genomic RNAs, and specific antiviral amiRNAs deficient in complementarity to the host sequences can be designed (García and Simón-Mateo, 2006). Moreover, antiviral amiRNA has less chance of recombining with or complementing non-target viral genome sequences and generating new viral pathogens (Gaffar and Koch, 2019). As amiRNAs are not recognized as the direct target of the viral suppressors, the amiRNA-mediated resistance is therefore more stable than the siRNA-mediated resistance (Duan et al., 2008).

MicroRNAs are molecules that are 21-24 nucleotides in length and commonly present in all eukaryotic organisms, which are responsible for regulating gene expression at the post-transcriptional level. Since their discovery in Arabidopsis (Llave et al., 2002), their role in plant biotic and abiotic stress management has been very crucial. MicroRNAs play key roles in regulating disease resistance signaling pathways, together with disease resistance-related gene expression, hormone signaling, and reactive oxygen species (ROS) production. They act as environmental response factors, inducing plants to overexpress or downregulate certain microRNAs or synthesize new amiRNAs in response to stresses, promoting plant evolution and adaptation.

The amiRNAs are intrinsically inscribed small RNAs produced from an immature base complemented hairpin prototype. These amiRNAs modulate gene expression by turning the expression of the target genes on/off or instigating the translation repression of the marked mRNAs (Meyers and Axtell, 2019). The amiRNA approach uses an intrinsic prototype amiRNA backbone where the nucleotide sequence of amiRNA can be substituted with complemented nucleotide sequences to the marked sequence (Ossowski et al., 2008). This strategy has been efficiently applied for downregulating the expression of endogenous genes in many plant species such as Arabidopsis, rice, bread wheat, tomato, eggplant, potato, tobacco, corn, barrel medic, soybean, and black cottonwood (Zhang et al., 2018). As seen in transgenic plants, the amiRNA strategy using different regions/genes has been proven to be successful against different begomoviruses such as tomato leaf curl New Delhi virus (ToLCNDV), TYLCV, and cotton leaf curl Burewala virus (CLCuBurV) (Ali et al., 2013; Sharma and Prasad, 2020) etc.

In this study, we designed a strategy to adopt an amiRNA-based approach using the intrinsic prototype of amiRNA-based constructs, namely, Rep-amiRNA1 and Rep-amiRNA2, for the production of whitefly-transmitted TYLCV-OM-resistant tomato plants. These transgenic tomato plants showed upregulated expression of amiRNA, which effectively downregulated or silenced the AC1/Rep transcripts of TYLCV-OM. This is first study using amiRNA to silence the AC1/Rep transcripts of TYLCV-OM infecting tomato plants in Oman.

## 2 Materials and methods

### 2.1 Design and construction of amiRNA expression constructs

The sequences of TYCLV-OM isolates and the replication initiator protein infecting the tomato crop were extracted from GenBank (accession number DQ644565). The conserved region with the Rep gene, amino acid, and nucleotide sequence analysis of different TYLCV-OM isolates were carried out using DNASTAR software. The WMD3 program was applied to design 22-nucleotide (nt) amiRNA sequences against the most conserved region of the AC1/Rep gene at the N-terminal end, encompassing nt 85 to 107. This position has been found to have certain domains that are involved in oligomerization, DNA binding, and DNA cleavage functions. The introduction of a mutation in this domain leads to the inactivation of the domain, resulting in the inhibition of viral replication. Two Rep-amiRNA1 (5′-AAGGTTGCATTCTTGAGAGCCC-3′) and Rep-amiRNA2 (5′-TTCCAACGTAAGAACTCTCGGG-3′), which are highly conserved for the TYLCV-OM Rep gene, were selected for cloning. In order to ensure any off-target effects, the 22-nt sequences were assessed against the tomato genomes, and the secondary structures of intrinsic amiRNAs were observed using MFOLD program (http://www.unafold.org/mfold/applications/rna-folding-form.php) by applying default algorithms. The cloning vector, pUC19-harboring amiRNAs were digested separately using the *Hind*III/*Eco*RI restriction sites. The cleaved amiRNA sequences were ligated at the *Hind*III/*Eco*RI site in the pCAMBIA2300 expression vector driven by a 35S cauliflower mosaic virus (CaMV) promoter and *nptII* terminator. The ligation reaction contained 1x T4 DNA Ligase buffer, 5 units of T4 DNA ligase (Invitrogen Inc., NY, USA), 100 ng digested pCAMBIA2300 vector, and 100 ng of amiRNAs and ddH_2_o. The intrinsic amiRNAs with a vector were transformed into the AGL1 strain of soil-borne *Agrobacterium tumefaciens* accompanied with 50 mg/L kanamycin and carbenicillin antibiotics.

### 2.2 Transient expression of amiRNA in *Nicotiana benthamiana* plants

The transient expression of amiRNA was studied in *Nicotiana benthamiana* plants using the leaf disc method through *Agrobacterium*-mediated infiltration, as described earlier (Ammara et al., 2015). A single colony of *A. tumefaciens* (AGL1 strain) harboring the amiRNA gene was selected from a fresh plate and transferred to the selective medium containing 200 µg/L carbenicillin and 50 µg/L kanamycin, incubated at an optimal temperature of 28°C with moderate shaking at 200 rpm for 48 hours. Once the optimal density (OD_600_) of bacterium was achieved, the bacterial cells were propagated in 10 ml MgCl_2_ (10 mM) and acetosyringone (100 mM) incubated at 25-28°C for 3-4 hours before inoculation. The inoculum was subverted into the leaves of *N. benthamiana* from the underside by using a 5/10 ml needleless injection syringe, as reported earlier (Saeed et al., 2018; Shafiq et al., 2021). The inoculated plants were maintained in an insect-free glasshouse with 16/8 hours day/night at 28°C. At 4-5 days post-inoculation (dpi) with the amiRNA gene, the inoculated *N. benthamiana* leaves were agroinoculated with TYLCV-OM, and all the plants were continuously being examined for symptom development.

### 2.3 Genetic transformation of tomato plants with amiRNAs

In plant genetic transformation experiments, tomato seeds, namely, Moneymaker, were surface sterilized with 70% ethanol for 1 min followed by treatment with 0.5% commercial bleach (Clorox) supplemented with 0.01% Tween-20 for 15 min; they were subsequently rinsed with sterilized dH_2_O for 5 min three times. The surface-sterilized seeds were transferred to a glass jar containing a seed germination medium (1x MS, 30 g/L sucrose, 0.5x Gamborg’s vitamins, 0.8% agar; pH 6.0). The seeds were germinated *in vitro* at 25°C with a 16/8 hours photoperiod (light/dark). After 9 days of seed germination, the cotyledons were cut at both edges using a sterilized scalpel blade. The cotyledons were also cut near the proximal end and distal end when they were sunken in the MSO. The cotyledons were placed on pre-culture medium (1mM Putrescine, 1 µg/L Zeatin Riboside, 0.1 µg/L IAA, 1x MS, 30 g/L sucrose, 1x Gamborg’s vitamins, 0.8% agar; pH 5.8). Before transformation, the explants were kept on a pre-culture medium in the dark at 25°C for 2 days. The pre-cultured explants were co-cultivated with *A. tumefaciens* harboring the pCAMBIA2300-amiRNA, supplemented with carbenicillin (200 µg/L) and kanamycin (50 µg/L) at 28°C for 2 days. The cotyledons from the pre-culture plates were picked with autoclaved forceps and were dipped in the *Agrobacterium* culture for 30 minutes with occasional changing of the side. The explants were shifted to sterile filter paper for drying. The cotyledons were then placed onto plates containing a co-culture medium (1mM putrescine, 1.0 µg/L zeatin riboside, 0.1 µg/L IAA, 200 mM acetosyringone, 1x MS, 30 g/L sucrose, 1x Gamborg’s vitamins, 0.8% agar; pH 5.8) for 2 days at 25 ^°^C. The control cotyledons without *Agrobacterium* treatment were also placed on a separate co-culture medium plate. After co-cultivation, the tomato cotyledons were transferred onto a plant selection medium (1mM putrescine, 0.5 µg/L zeatin riboside, 0.05 µg/L IAA, 250 µg/L cefotaxime, 50 µg/L kanamycin, 1x MS, 30 g/L sucrose, 1x Gamborg’s vitamins, 0.8% agar; pH 5.8). The cotyledons were kept on a selection medium, with transferal to a fresh selection medium occurring at every two weeks until calluses were formed. The control cotyledons were also kept on a selection medium with or without antibiotics. The shoots that regenerated from the calluses were carefully excised with a sterilized blade, with some calluses on the base, and transferred onto a shoot elongation medium (1mM putrescine, 0.5 µg/L BAP, 250 µg/L cefotaxime, 25 µg/L kanamycin, 0.5X MS, 30 g/L sucrose, 1x Gamborg’s vitamins, 0.8% agar; pH 5.8) in baby food jars. The shoots were transferred every 15 days to a freshly prepared shoot elongation medium. When the shoots became 4-5 cm long, they were carefully removed from the shooting medium and transferred to a rooting medium (1mM putrescine, 1.0 µg/L IBA, 50 µg/L kanamycin, 0.5x MS, 30 g/L sucrose, 1x Gamborg’s vitamins, 0.8% agar; pH 5.8). The plantlets with root growth were removed from the jar, washed gently under tap water to remove residual agar, and were transferred to plastic pots containing a sterilized wet soil mix (peat moss + compost + sand 1:1:1). The pots were covered with polythene bags to maintain high humidity and were kept at 28 ^°^C. After 3-4 days, the polythene bags were punctured in order to slowly reduce the humidity, followed by the removal of the polythene covers after one week. When the plants reached a reasonable size after two months, they were shifted to bigger plastic pots (12″ wide x 11″ tall) filled with the soil mix and kept under a glasshouse at 28 ^°^C.

### 2.4 Molecular analysis of F0 (transgenic) tomato plants

The total DNA was isolated from the T0 transgenic plants at 30 dpi using the CTAB method, as described previously (Doyle, 1990). The total DNA was used as a template for the PCR amplification of the amiRNA gene using 35S CaMV promoter-specific primers: FD-35S (5’-ACGACACTCTCGTCTACTCCAAGA-3’) and RD-35S (5’-TGCTTTGAAGACGTGGTTGGAACG-3’). The amplicons (300 bp) were visualized on 0.8/1.0% (w/v) agarose gel under UV illumination and photographed. The virus accumulation was further confirmed by Southern blot hybridization using a DIG-labeled probe kit. To detect the amiRNA gene in the transformed tomato lines, a coat protein (CP)-based DNA probe was developed and labeled with digoxigenin-11–dUTP using DIG-High Prime kit I (Roche), as per the manufacturer’s directions. The hybridized membrane was washed with a saline–sodium citrate (SSC) buffer consisting of different percentages of sodium dodecyl sulfate (SDS) at various temperatures. After hybridization, the membrane was incubated in a blocking buffer followed by the addition of an antibody solution. Finally, the membrane was treated with an NBT/BCIP chromogenic substrate solution, which revealed distinct color bands upon incubation for 4/6 hours in the dark.

### 2.5 Infectivity analysis of F1 (transgenic tomato) plants

To assess the susceptibility to infection of F1 plants, an infectious construct of the TYLCV-OM genome was produced following the protocols described earlier (Amrao et al., 2010). Initially, a segment of ~1.9kb DNA covering the intergenic region (IR) was released from the pUC19-TYLCV monomer DNA molecule with *Pst*I and *Eco*RI restriction endonucleases, and it was ligated into the pCAMBIA-1301 binary vector at the *Pst*I/*Eco*RI sites, generating a pCAM-1.9TYLCV construct. In the second step, a full-length monomer molecule of TYLCV-OM (approximately 2.7 kb) was released with the *Pst*I restriction enzyme and was ligated into pCAM-TYLCV at the *Pst*I site. The confirmed infectious construct was transformed into *A. tumefaciens* strain AGL1, accompanied by 50 mg/L each of kanamycin and carbenicillin antibiotics. An optical density (OD_600_) of 0.8-1.0 was achieved at 28 °C for 48 hours, and a bacterium solution containing 3 mL was mixed with 10 mM MgCl_2_ and 100µM acetosyringone and incubated at 28 ^°^C for activation of the bacterial cells. To test resistance in transgenic tomato plants, the tomato seedlings transgenic line integrated with the amiRNA genes and a non-transgenic-control plant at the 3-4 leaf stage were agroinfiltrated with activated *A. tumefaciens* containing pCAM-TYLCV (infectious virus construct). The inocula were injected at the lower side of the leaves using 5/10 mL needless injection syringes. The non-transformed susceptible tomato was also agroinfiltrated with only agrobacterium strain AGL1 as a mock control. The agroinoculated plants were maintained in a glasshouse at 28 ± 2 °C, photoperiod 16/8 hours, and 60-70% humidity, and symptom appearance was perceived until 70 days. Individual agroinfiltrated plants were evaluated using the disease scoring scale described by Al-Shihi et al. (2018).

### 2.6 Molecular analysis of transgenic tomato (F1) plants

The total DNA was extracted from the transgenic and non-transformed plant leaves at 30 dpi separately following the prescribed method. To detect TYLCV in the agroinfiltrated plants, PCR diagnosis was implemented with diagnostic primers specified for TYLCV detection (Ammara et al., 2015). Moreover, virus accumulation in agroinoculated plants was validated by the Southern hybridization blotting method, as described in the previous section. Furthermore, the presence of virus particles in inoculated transgenic and non-transgenic plants was confirmed by ultrastructure studies. Plant samples preparation for transmission electron microscopy (TEM) included dissection, dehydration, infiltration, embedding, and polymerization. The samples were processed with primary fixation in 2.5% glutaraldehyde solution (Karnovsky’s fixative) prepared in 0.1M cacodylate, and they were stored at 4 °C. To stop fixation, the samples were washed (2X) in a 0.1M cacodylate buffer (pH 7.4) for 10 min at 4 °C. The samples were post-fixed in 1% osmium tetroxide for 1 hour at room temperature, followed by 2X washing in distilled water twice for 10 min each. After fixation, the samples were dehydrated (15-30 minutes/change) with different concentrations of acetone: 30%, 50%, 70%, 80%, 90%, and 100%. The dehydration was continued with two more changes: (1) with 100% acetone (30 min per change) followed by (2) 100% acetone with one 30 min change of 100% acetone:propylene oxide (1:1). The samples were infiltrated in 1:1 resin-to-acetone mixture for 1 hour, followed by 3:1 resin-to-acetone mixture for 30 min, then in absolute resin for 1 hour. Afterward, the samples were embedded in pure Araldite resin in beam capsules and polymerized at 60 ^°^C overnight, producing resin blocks containing the sample. The blocks were sectioned for light microscopy using glass knives in an Ultracut E Reichert–Jung ultramicrotome. Sections of 500 nm thickness were placed on a glass slide, after which they were allowed to dry, stained with toluidine blue, and viewed under the light microscope; sections were then selected for TEM microscopy. The blocks were finely trimmed and sectioned at 70 nm thickness using glass knives in the Ultracut E Reichert–Jung ultramicrotome. The sections were stained with uranyl acetate, followed by lead citrate, and then the ultrathin sections were viewed using TEM.

## 3 Results

### 3.1 Design of vector-generating amiRNAs

**Figure 1:**
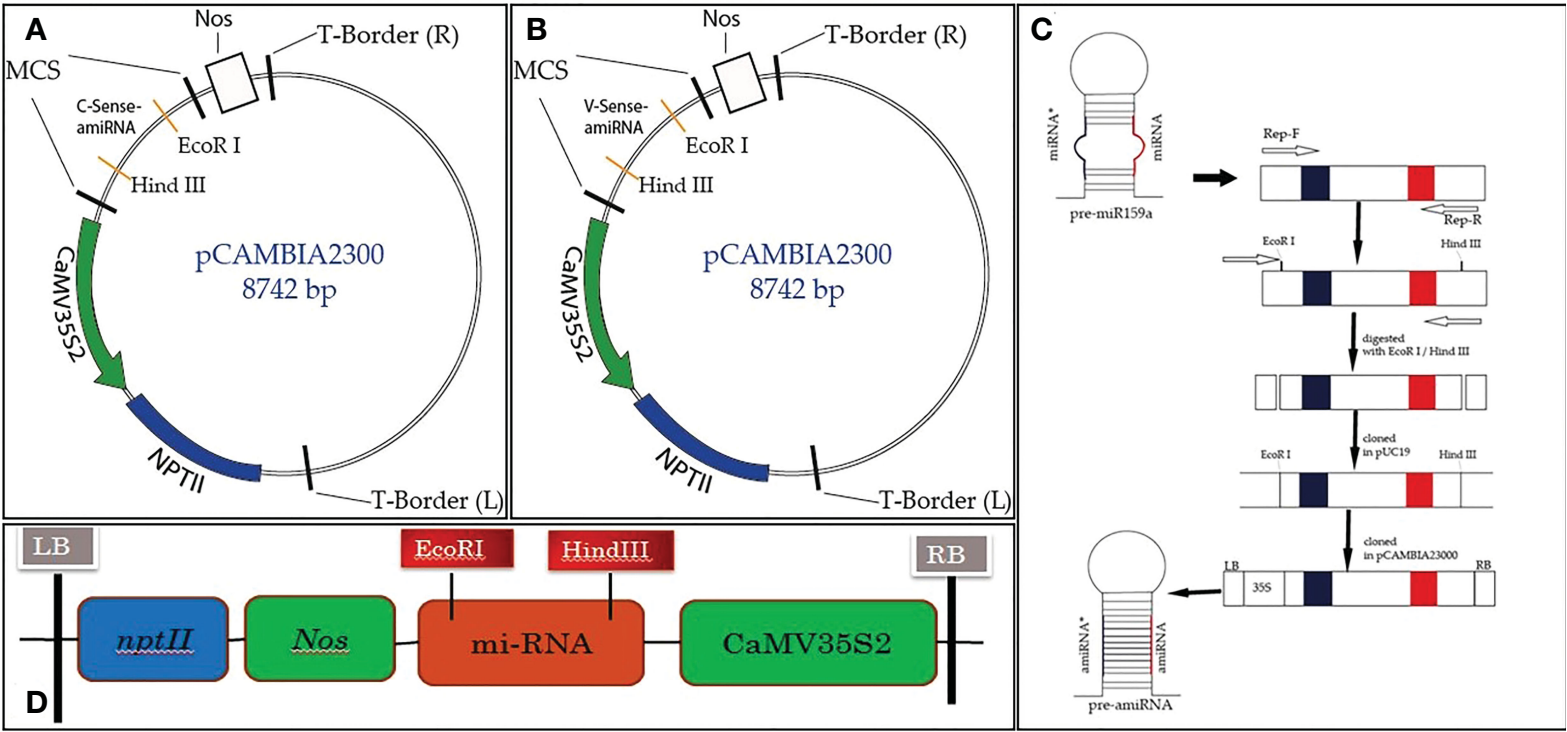
Schematic diagram of Rep-amiRNA1 **(A)** and Rep-amiRNA2 **(B)** cloned at the *EcoR*I/*Hind*III site of the pCAMBIA2300 plant expression vector, comprising the 35S2 promoter, *Nos* terminator, and *Npt*II as selectable markers **(C)**.

**Figure 2:**
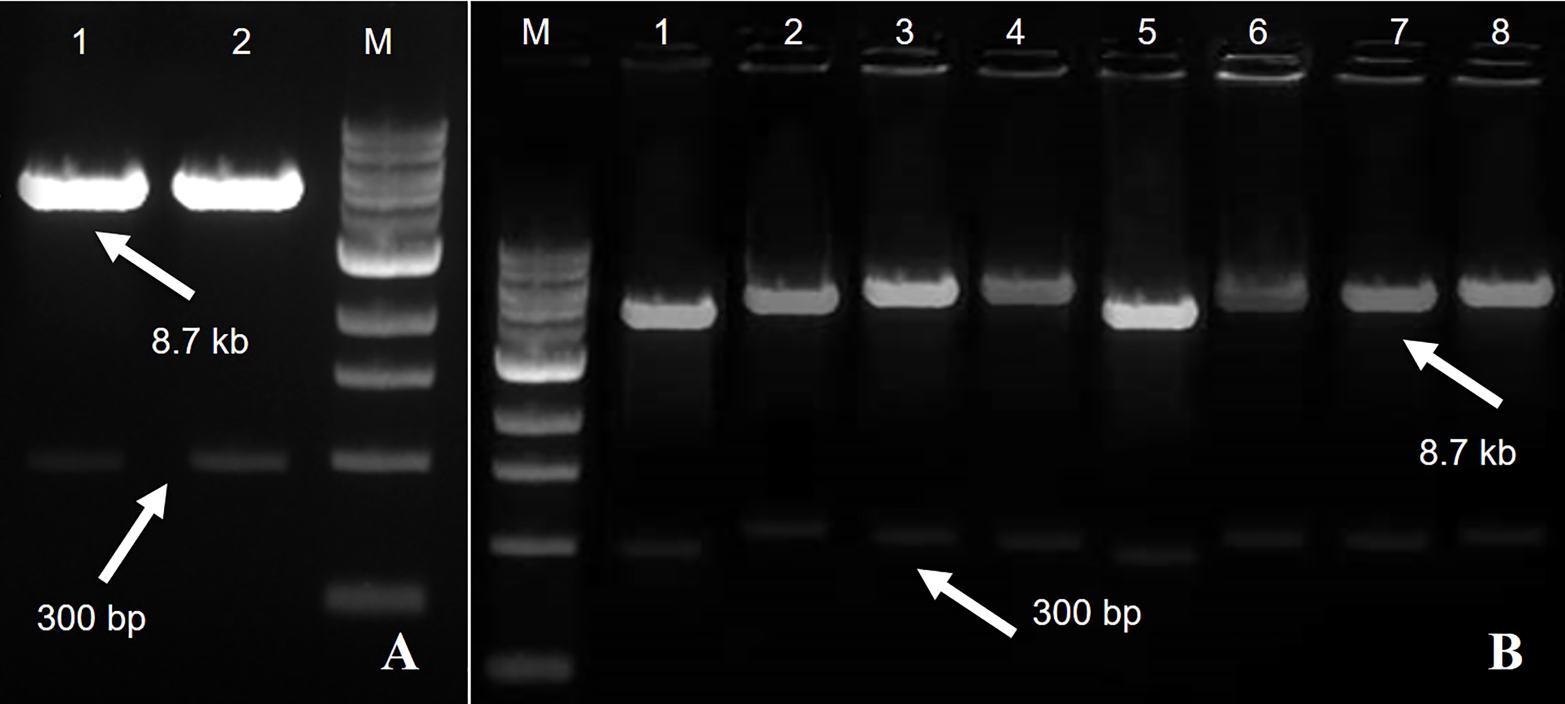
A fragment of 8.7 kb of expression vector pCAMBIA2300 Rep-amiRNA1 (Lane 1) and Rep-amiRNA2 (Lane 2) with a size of 300 bp. M= DNA marker **(A)**. *Agrobacterium* transformed with pCAMBIA2300 harboring Rep-amiRNA1 (Lane 1-4) and Rep-amiRNA2 (Lane 5-8). A fragment of 8.7 kb and 300 bp was yielded. M=DNA marker **(B)**.

The TYLCV-encoded AC1/Rep gene plays an important role in viral replication and pathogenicity. For this reason, it is the best candidate for imparting resistance in plants *via* the amiRNA strategy. To achieve the expected results, the sequence (nucleotide and amino acid) of the AC1/Rep gene was analyzed/compared with all the isolates of the TYLCV-OM strain causing yellow leaf curl disease in the tomato crop. The multiple sequence alignment exhibited the position of the ATP/GTP-binding motifs of the Rep gene of TYLCV, which served the purpose of designing two predicted amiRNAs (Rep-amiRNA1 and Rep-amiRNA2) that target the ATP/GTP-binding motifs, which were successfully generated by targeting the most conserved domain of the Rep gene. The amiRNA sequences were replaced with the original 22-nucleotide miR159a sequence in the miR159a backbone. Afterwards, the resultant microRNA (Rep-amiRNA1 and Rep-amiRNA2) constructs were successfully cloned into a pCAMBIA2300 plant expression vector driven by a 352S CaMV promoter and *nptII* terminator at compatible sites, and they were transformed into an *A. tumefaciens* AGL1 strain (**Figures 1**, **2**).

### 3.2 Transient expression of amiRNA constructs

**Figure 3:**
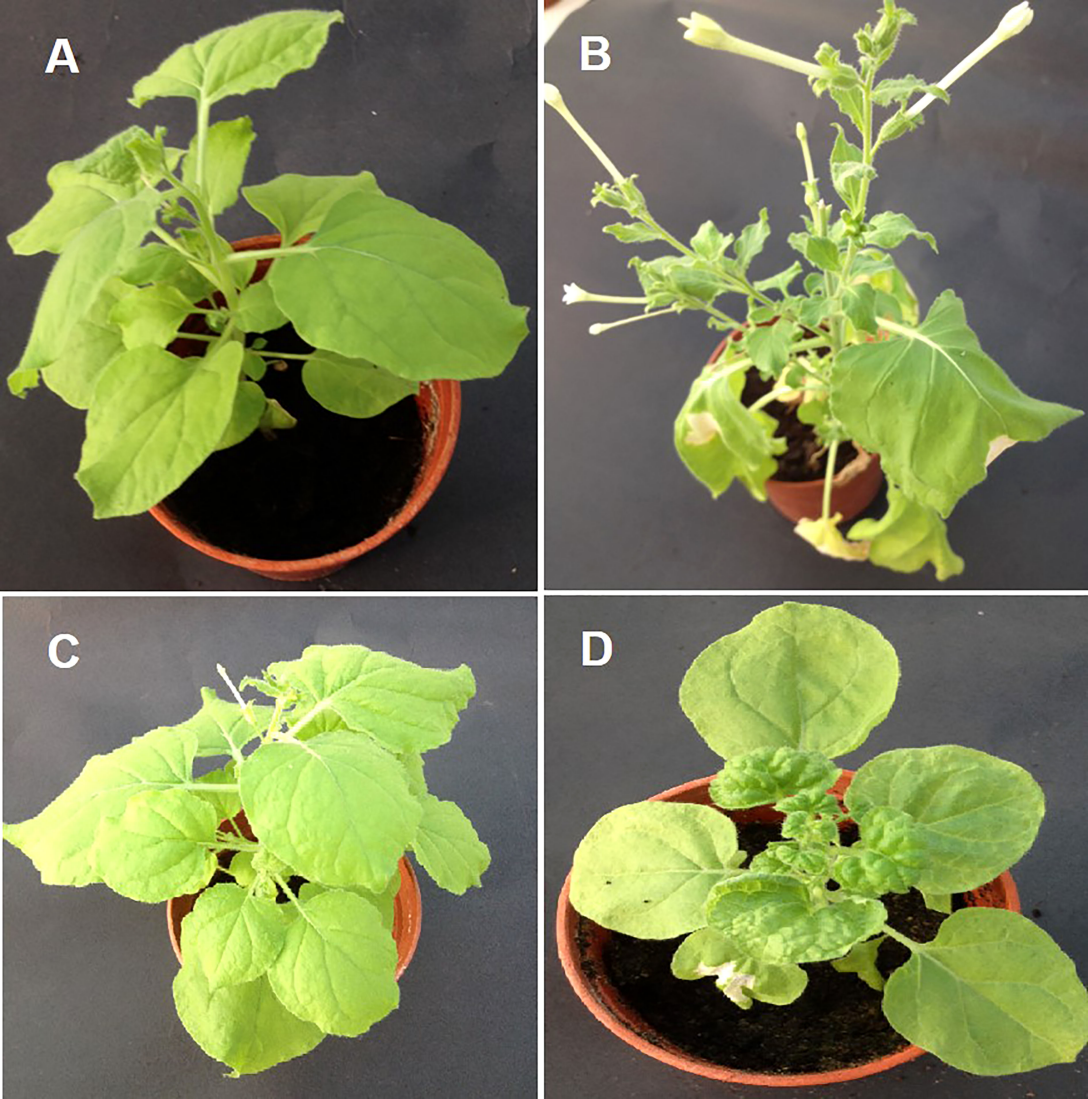
Transient assay with Rep-amiRNA1 and Rep-amiRNA2 constructs in *N. benthamiana* plants agroinfiltrated with Rep-amiRNA1 **(B)** and Rep-amiRNA2**(C)**. **(A)** is a healthy plant, shown here in comparison with a symptomatic plant **(D)**.

**Figure 4:**
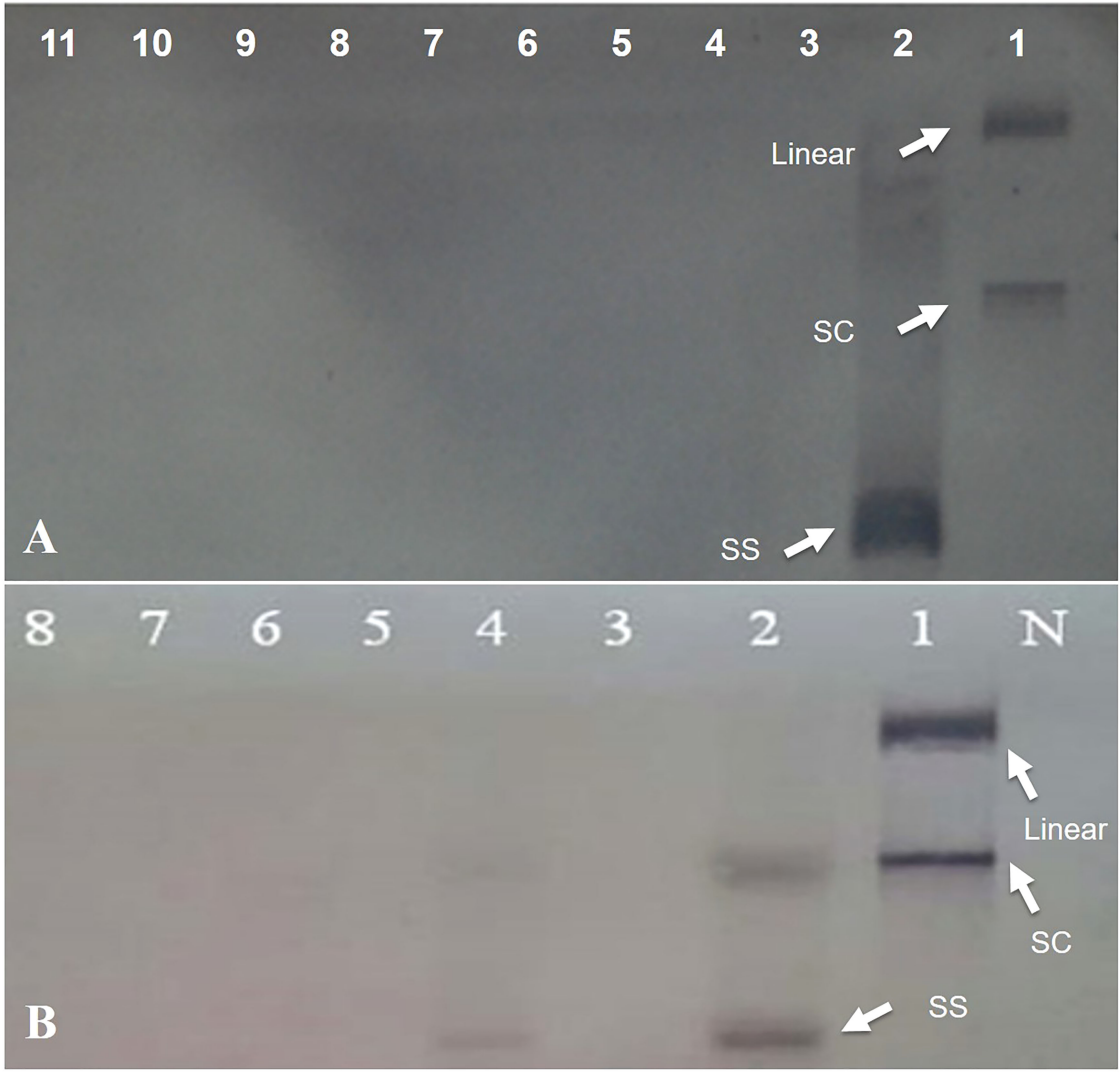
Southern hybridization blotting investigation of *N. benthamiana* plants inoculated with Rep-amiRNA1 (Lane 3-7) and Rep-amiRNA2 (Lane 8-11) and challenged with TYLCV. DNA from symptomatic plant inoculated with TYLCV only (Lane 2). Lane 1 is plasmid DNA. The position of single-stranded (ss), supercoiled (sc), and linear (lin) viral DNA forms is indicated with arrow. N= Negative control **(A)**. Southern blot analysis of F1-transformed tomato plants inoculated with Rep-amiRNA1 (Lane 4-8) and Rep-amiRNA2(Lane 3) and challenged with TYLCV-OM. DNA from symptomatic plant inoculated with TYLCV-OM as control (Lane 2). Lane 1 is plasmid DNA. N= Negative control. The position of single-stranded (ss), supercoiled (sc), and linear (lin) viral DNA forms is indicated with arrow **(B)**.

To assess the effectiveness of the designed amiRNA in silencing the AC1/Rep gene, *in vivo* screening assays were performed. An *Agrobacterium*-mediated co-transformed assay was conducted where the cells containing Rep-amiRNA1 and/or Rep-amiRNA2 were transiently expressed in *N. benthamiana* plants. Typical symptoms of TYLCV infections exhibiting as yellowing, stunting, leaf curling, and necrosis were observed in only TYLCV-infected plants at 21 dpi, and none of the viral symptoms were observed on the mock inoculated *N. benthamiana* plants. Nevertheless, the *N. benthamiana* plants agroinfiltrated with Rep-amiRNA1 or Rep-amiRNA2 showed mild symptoms (slight yellow and mild curling) in 1 and 2 N*. benthamiana* plants, respectively. Overall, the plants agroinoculated with Rep-amiRNA1 showed a healthier effect compared to the plants inoculated with the Rep-amiRNA2 construct (**Figure 3**). These results were further confirmed by PCR analysis, where 40% of the *N. benthamiana* plants (**Table 1**) showed the desired amplification of 650 bp with CP-specific primers. The *N. benthamiana* plants inoculated with Rep-amiRNA1 (**Supplementary Figure 1**, Lane 8) and Rep-amiRNA2 (**Supplementary Figure 1**, Lanes 13 and 14) showed strong bands, followed by Lanes 4, 5, 7, 12, and 15 exhibiting faint bands and lanes (**Supplementary Figure 1**, Lanes 1, 2, 3, 6, 9, 10, 11, 16, 17 and 18) that did not show any bands in gel electrophoresis imaging, indicating that the amiRNA constructs prevented the spread of the virus from inoculated tissues. Southern blot hybridization of viral DNA from randomly selected *N. benthamiana* plants inoculated with Rep-amiRNA1 and Rep-amiRNA2 was performed using a TYLCV-OM-specific DIG-labeled probe. No hybridization was observed in the inoculated plants except in positive control. This result indicates that the replication and accumulation of the virus in the plants inoculated with Rep-amiRNA1 and/or Rep-amiRNA2 is undetectable (**Figure 4A**); hence, these Southern blot hybridization results support the PCR results. Since the performance of both the constructs was not significantly variable, it was decided to use both the constructs (Rep-amiRNA1 and Rep-amiRNA2) for tomato cv. Moneymaker for plant genetic transformation.

**Table 1:** Symptoms severity scoring of Rep-amiRNA1 and Rep-amiRNA2 in transient expression of N. benthamiana plants.

Inoculum	#Exp.	Plant infectivity (infected/inoculated)			SS*	Resistance %	Detection
		8dpi	16dpi	24dpi			PCR/ Southern blotting
Rep-amiRNA1+ TYLCV-OM	I	0/5	0/5	1/5	2	80	1/5
	II	0/5	0/5	1/5	0	80	1/5
Rep-amiRNA2+ TYLCV-OM	I	0/5	1/5	2/5	1	60	2/5
	II	0/5	0/5	1/5	2	80	1/5
TYLCV-OM	I	3/3	3/3	3/3	4	–	3/3
	II	3/3	3/3	3/3	4	–	3/3
MC**	I	0/3	0/3	0/3	0	NA	0/3
	II	0/3	0/3	0/3	0	NA	0/3
NI***	I	0/2	0/2	0/2	0	NA	0/2
	II	0/2	0/2	0/2	0	NA	0/2

*Severity of symptoms using scale as described by Adel Al-Shihi et al. (2018).

**N. benthamiana plants agro-infiltrated with binary vector only.

****Non-inoculated N. benthamiana plants.NA and - , Not applicable.

### 3.3 Transformation of tomato plants with amiRNA constructs

**Figure 5:**
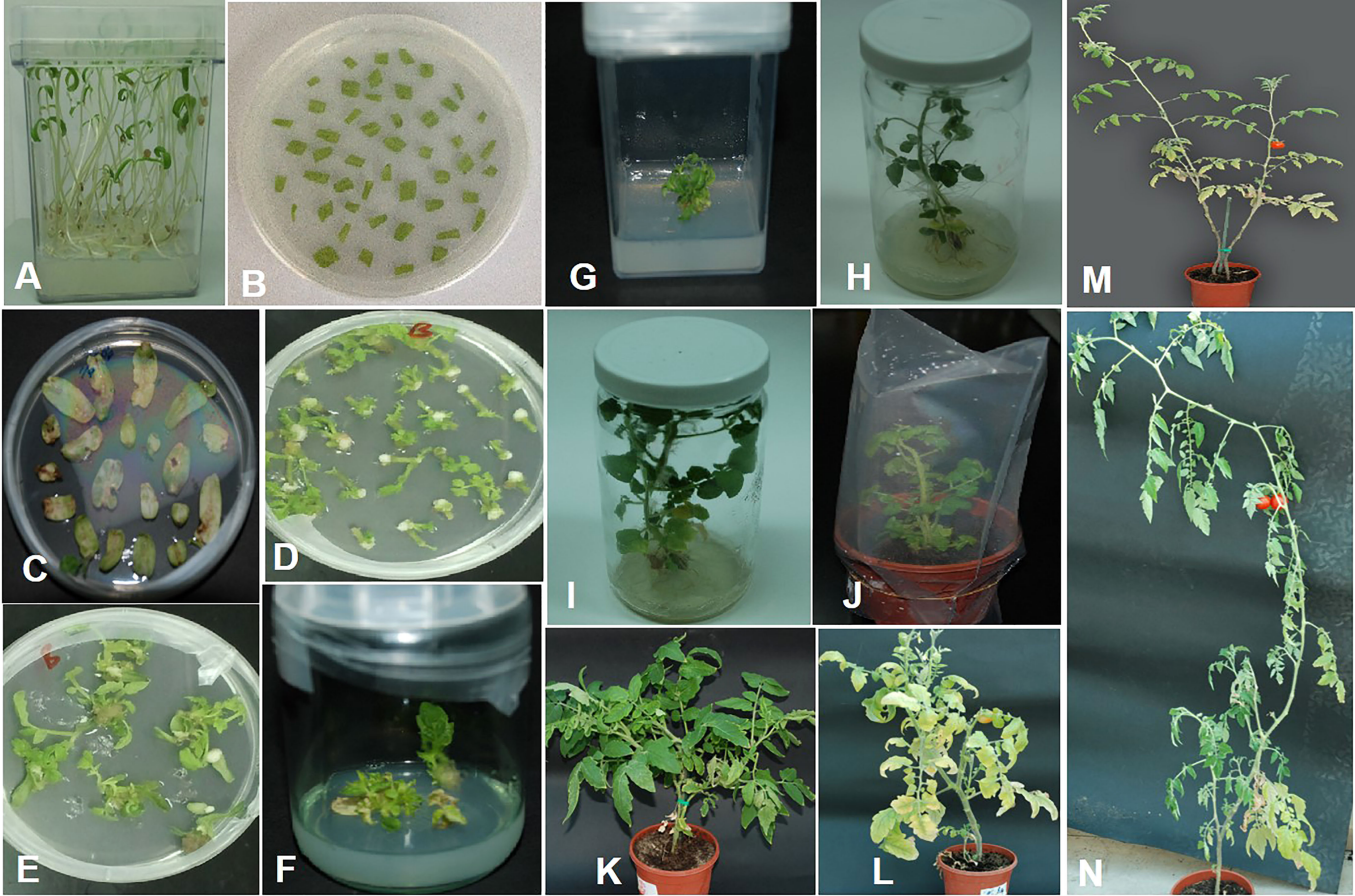
Tomato transformation. **(A)** Seedlings of tomato germinated on MS medium. **(B)** Cotyledon explants on pre-culture medium. **(C)** Cotyledon explants on selection medium after co-cultivation with *Agrobacterium*. **(D, E)** Callus with regeneration shoots on selection medium. **(F)** Shoots regenerating from a single callus growing on selection medium. **(G)** Cotyledon explant was discarded. **(H)** Young plantlet on shoot elongation medium. **(I)** Plantlet with well-developed roots on rooting medium. **(J)** Fully regenerated plant hardened in soil. **(K)** Two months after hardening. **(L)** Plant at flowering stage. **(M, N)** Mature transgenic tomato plants with fruits.

The tomato var. Moneymaker transgenic lines were generated, as discussed earlier (Ammara et al., 2015), with slight modifications. In the initial step, tomato seeds were sown on a germination medium, and explants from nine-day-old seedlings were cultured on pa re-culture medium appended with 1 mM putrescine, 1 µg/L zeatin riboside, and 0.1 µg/L IAA for 48h. *Agrobacterium* carrying Rep-amiRNA1 or Rep-amiRNA2 recombinant construct was used for transformation of the explants. Co-cultivation of the explants in bacterial suspension with OD_600_ of 1.0 (corresponds to 1.0×10^8^ cells/ml) for 30 min was found to give high transformation efficiency. The co-cultivated explants were cultured on a co-culture medium containing 1.0 µg/L zeatin riboside, 0.1 µg/L IAA, and 200 mM acetosyringone. Acetosyringone enhances *Agrobacterium* infection and the T-DNA delivery process. The inhibitory effects of the antibiotic cefotaxime were used to control excessive growth of *Agrobacterium*. The explants were washed with different concentrations of cefotaxime (250 µg/L, 500 µg/L). Almost 81% of the co-cultivated explants (number of explants grown on kanamycin containing a medium/total number of co-cultivated explants) grew on a selection medium containing 0.5 µg/L zeatin riboside, 0.05 µg/L IAA, 250 µg/L cefotaxime, and 50 µg/L kanamycin. The non-transformed explants turned brown, and excessive growth of *Agrobacterium* was observed. The non-transformed explants were discarded. A total of 72% of the explants started to produce green calluses two weeks after co-cultivation. Only 5-10% of the explants showed slow growth of the callus and shoots, and explants that lost regeneration ability were discarded. Two to five shoots per explant/callus were produced three to six weeks after co-cultivation. Regenerating shoots were cut from the explant/callus and transferred to the same medium. Each explant/callus represents one independent line, and each shoot represents one replicate. The regeneration rate of the transformed explants was low in comparison to the control plants. Eight weeks after shoot development, the cotyledon explant was cut from the growing shoots and discarded. The regenerating shoots were allowed to proliferate and elongate by shifting to a shoot elongation medium supplemented with 0.5 µg/L BAP, 250 µg/L cefotaxime, and 25 µg/L kanamycin. After three to five weeks of culture on a shoot elongation medium, elongation of shoots in 90% of the regenerating shoots was observed. A small number of healthy shoots showed a slow response in the shoot elongation medium. At the stage of 7-9 cm long and 5-6 leaves, newly developed plantlets were moved to the rooting medium accompanied by 1.0 µg/L IBA and 50 µg/L kanamycin. After two to four weeks of culturing on the rooting medium, 85% of the shoots developed hairy roots. The shoots that did not show any root growth after eight weeks of transferal to the rooting medium were thrown away. The fully regenerated plants were hardened in soil in small pots, and a survival rate of 92% was recorded (**Figure 5**).

### 3.4 Verification of amiRNA genes in virus-inoculated transgenic tomato plants

**Figure 6:**
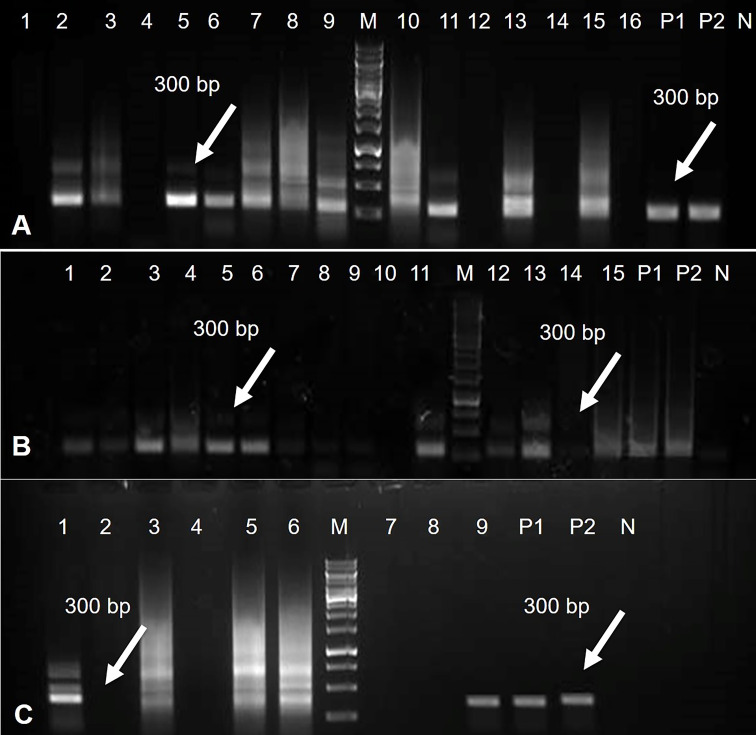
PCR results for Rep-amiRNA1 construct amplified from transformed tomato plants. Amplicon of 300bp was observed in 11 plants transformed with Rep-amiRNA1 construct. P1, P2= Positive control, N= Negative control, M= 1kb marker DNA **(A)**. PCR products were analyzed for Rep-amiRNA1 and Rep-amiRNA2 constructs amplified from transformed tomato plants. Amplicon of 300bp was observed in 10 plants transformed with Rep-amiRNA1 (Lane 1-9, 11) and three plants transformed with Rep-amiRNA2 (Lane 12, 13, 15). P1, P2= Positive control, N= Negative control, M= 1kb marker DNA **(B)**. PCR products were analyzed for Rep-amiRNA1 and Rep-amiRNA2 constructs amplified from transformed tomato plants. Amplicon of 300 bp was observed in three plants transformed with Rep-amiRNA1 (Lane 1, 3, 9) and two plants transformed with Rep-amiRNA2 (Lane 5, 6). P1, P2= Positive control, N= Negative control, M= 1kb marker DNA **(C)**.

The tomato transgenic lines were successfully transformed with the Rep-amiRNA1 (83%) and Rep-amiRNA2 (16.6%) constructs. All transformed plants were confirmed by PCR for the presence of 35S promoter, and an amplification of 300 bp was obtained in 72% and 14% of the transformed plants with Rep-amiRNA1 and Rep-amiRNA2 genomes, respectively (**Figures 6A–C**). Transformation competence was determined as the percentage (%) of plants found to be PCR positive. The non-transformed plants were discarded from the experiment, and out of all the transformed plants, 68% of them started flowering three months after transfer to soil, whereas only 28% of the plants developed fruits and seeds that were collected after three to four months of planting (**Figure 5** (L, M, N). Uncontaminated transgenic tomato plant (T0) seeds were collected and stored at 4 °C. Five to six seeds for each independent line were sown in a seedling tray filled with a sterilized potting mix, which was purchased commercially. Four-week-old tomato seedlings were transferred to plastic potted soil, the total nucleic acid was extracted, and the integration of the amiRNA genes into the F1 transgenic plants was confirmed by a PCR assay, which yielded the expected amplification and validated the integration of the amiRNA gene into the F1 lines (**Supplementary Figure 2**).

### 3.5 Assessment of resistance of T1 transgenic tomato plants to TYLCV

To investigate the ability of the Rep-amiRNA to express resistance against TYLCV-OM, the non-transgenic (susceptible) and transformed lines (F1) were targeted with TYLCV–OM at the 3 to 4 leaf stage through agrobacterium-mediated inoculation. The TYLCV-OM agroinoculation assay was performed, and disease scoring was also carried out. At 21 dpi, emergence of disease symptoms (yellow leaf curl) was noticed (rating 1 at disease scoring scale), which later progressed to severe symptoms (rating 3 at disease scoring scale) at 30 dpi in non-transgenic tomato plants, whereas no symptoms were observed on transgenic plants where all the plants’ newly emerged leaves showed a healthy growth (**Figures 5K–N**). Nevertheless, only mild disease symptoms (rating 1 at disease scoring scale) were observed in one Rep-amiRNA1 transgenic plant at 60 dpi. Furthermore, to confirm the symptom development at a later stage, all F1 transgenic tomato lines were observed until 70 dpi, and none of the transgenic plants exhibited any obvious symptom development. The transgenic plants were propagated normally as wild-type plants, and they started flowering and bearing fruits, which were collected and stored for further analysis. The F1/non-transgenic tomato plants were analyzed for the detection of virus particles using TYLCV-specific primers, as described earlier. The expected virus DNA fragment of 650 bp was amplified in the non-transgenic plants, and there was only one from the F1 plants that showed mild symptoms (**Figure 4** BL14). However, the rest of the samples tested negative for virus infection, suggesting amiRNA gene confer resistance against TYLCV-OM that silenced the viral gene in the transgenic plants (**Supplementary Figure 3**). Furthermore, to evaluate the viral accumulation with symptoms expression in the transgenic and non-transgenic plants, Southern blotting was performed by probing with the CP gene. The hybridization results demonstrated that transgenic plants containing amiRNA constructs were overexpressed, which notably reduced accumulation of TYLCV DNA at 30 dpi, compared to the non-transgenic plants (**Figure 4B**), thus complementing the PCR findings. Subsequently, different replicative forms such as linear double-stranded DNA, supercoil (SC), and single-stranded (ss) were also recognized, compared with the digested plasmid DNA of TYLCV used as a control (**Figure 4B**). The hybridization results identified both SC and SS DNA forms in the non-transgenic plant (**Figure 4B** lane 2) compared with the virus genome control (**Figure 4B** lane 1). Moreover, the ssDNA form was detected in one PCR-positive transgenic plant (**Figure 4B** lane 4). Altogether, according to the results obtained from the molecular diagnosis and disease scoring, the resistance in the transgenic lines varies from 50% to 83%, which is notably higher compared to the available natural, tolerant genotypes against TYLCV, demonstrating that amiRNA-based resistance remarkably increases TYLCV resistance (**Table 2**).

**Table 2:** Response of F1 (transgenic tomato lines) harboring Rep-amiRNA1 or Rep-amiRNA2 agro-infiltrated with TYLCV-OM.

Transgenic line	TYLCV-OM (30 dpi)			TYLCV-OM (60 dpi)			TYLCV-OM (70 dpi)		
	Symptomatic/ inoculated	SS*	Resistance (%)	Symptomatic/ inoculated	SS*	Resistance (%)	Symptomatic/ inoculated	SS*	Resistance (%)
1	1/6	0-1	83	1/6	1	83	1/6	1	83
2	0/6	0	100	0/6	0	100	1/6	0-1	83
3	0/6	0	100	1/6	1	83	2/6	1	66
4	0/6	0	100	1/6	0-1	83	3/6	2	50
5	1/6	0-1	83	1/6	1	83	2/6	1	66
6	1/6	1	83	2/6	1	66	3/6	1	50
MC**	0/10	–	–	0/10	–	–	0/10	–	–
NT***	8/8	1	0	8/8	2	0	8/8	3	0

*Severity of symptoms using scale as described by Adel Al-Shihi et al. (2018).

**Non-transgenic tomato cv. money maker plants agro-infiltrated with expression vector only.

***Non-transgenic tomato cv. money maker plants agro-infiltrated with TYLCV Oman strain.

-, Not applicable.

**Figure 7:**
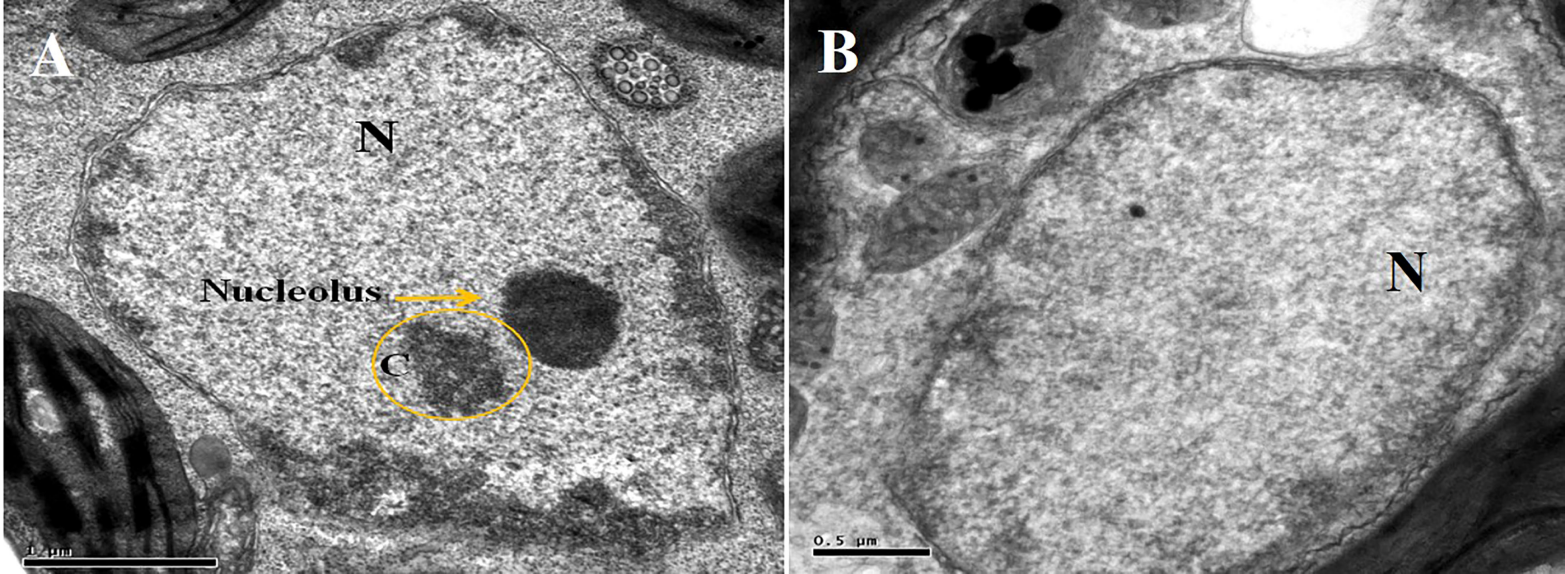
Electron micrograph of a phloem **(A)** and parenchymal **(B)** cells from agroinoculated, symptomless transgenic plant. Nucleolus is obvious in the nucleus “N”, and a small clump “C” is also visible.

Furthermore, TYLCV infection was also found to induce changes in the nuclei of the parenchymal cells of the phloem when using TEM. The nuclei of the infected cells were recognized by their granular structure, the presence of nuclear inclusions, and the apparent absence of nucleoli (**Figure 7B**). However, nuclei in the tissues of the symptomless inoculated transgenic plant were normal, though small clumps (C) were also observed (**Figure 7A**).

## 4 Discussion

Over the last two decades, several researchers have sought the application of pathogen-derived resistance for developing genetically engineered resistance against whitefly-transmitted geminiviruses. In the begomovirus genus, replication-associated genes (AC1/AC2/AC3) (Day et al., 1991; Brunetti et al., 2001; Ammara et al., 2015; Sharma and Prasad, 2020) and the CP gene (Kunik et al., 1994; Ammara et al., 2015) have been used to target monopartite begomoviruses. All these approaches were used to interfere either with RNA (based on gene silencing) or the protein of viral genes (based on negative dominant interference). However, not a single approach was capable of delivering resistance in the field, implying that those new tools may be advanced enough to improve resistance against begomoviruses. In an attempt to regulate infection by ssDNA viruses, the current transgenic approach is based on the expression of pathogen-derived double-stranded or hairpin RNAs that confer resistance to RNA and DNA plant viruses. For this purpose, a highly conserved replication initiator gene (C1/Rep) was selected to study Rep protein regulation in global transmitting TYLCV, which is prerequisite for the replication of all ssDNA viruses. Two amiRNA constructs were designed to target the Rep gene of TYLCV-OM to initiate resistance against this virus. Higher expression of amiRNAs was achieved, indicating the correct processing of pre-miR159a (Niu et al., 2006; Khraiwesh et al., 2008). For this reason, in this study the hairpin structure of miR159a was utilized for the construction of the amiRNAs. Amin et al. (2011) successfully exploited the amiRNA approach and detected a high level of miR159a expression in *N*. *benthamiana* plants upon infection by different cotton-infecting begomoviruses. In *Arabidopsis*, miR159a was found to target genes that play a role in the transition to the reproductive phase and initiation of flowers (Parcy et al., 2002). Consequently, pre-miR159a harboring the sequence of amiRNA with a high expression level was proposed to be correctly processed into mature amiRNAs and to induce silencing of the target gene. These amiRNAs targeted the region involved in DNA binding and cleavage domains located at the N-terminal end. Inactivation of these two domains leads to the prevention of viral replication. The sense and antisense transgenes would prevent expression of the target gene by promoting degradation of the target mRNA (Asad et al., 2003). Recently, it was shown that it is possible to develop resistance to TYLCV by introducing transgenes homologous to the Rep gene, and silencing of the Rep gene can occur through a mechanism of post-transcriptional gene silencing (PTGS) (Orozco and Hanley-Bowdoin, 1996). The multifunctional character of the Rep protein makes it a suitable target to develop resistance strategies (Orozco and Hanley-Bowdoin, 1996). It was shown that the expression of the Rep protein of tomato yellow leaf curl Sardinia virus (TYLCSV) lacking a C-terminal end resulted in TYLCSV resistance in tomato (Brunetti et al., 1997). Sardo et al. (2011) have used a C-terminal-truncated Rep transgene to develop TYLCSV resistance in *N*. *benthamiana*. Moreover, amiRNA has been successfully used to confer resistance against different viruses, including turnip yellow mosaic virus (TuYMV) and turnip mosaic virus (TuMV) (Niu et al., 2006), cucumber mosaic virus (CMV) (Qu et al., 2007; Duan et al., 2008), watermelon silver mottle virus (WSMoV) (Huang et al., 2015), and tomato leaf curl New Delhi virus (ToLNDV), as well as DNA virus (Sharma and Prasad, 2020). The transgenic plants from independent transformants showed a constitutive expression of the amiRNAs transgenes, which confirmed the successful transformation of amiRNA constructs into tomato plants through *Agrobacterium*. High expression of the green florescence protein (GFP) transgene coupled with CaMV 35S promoter was identified by the strong green fluorescence of the transformed tissues (Sunilkumar et al., 2002; Wang et al., 2008).

Niu et al. (2006) showed that amiRNA genes are dominant, and the resistance phenotype can be screened at F1 generation. They found that amiRNA constructs targeting the 21nt sequence of TuMV CP were very effective. Similar results are shown here in this study, where transgenic tomato plants carrying amiRNA constructs showed resistance to TYLCV-OM infection. Interestingly, Southern blotting displayed no or a very low level of TYLCV-OM replication, suggesting that amiRNAs are expressed at higher levels and could prevent the replication of the virus. However, the breakdown of resistance/mild symptoms and limited tolerance to TYLC was observed in one transgenic plant, suggesting that the level of transgene expression is not sufficient to silence the virus. This demonstrates the importance of transformation events, target site selection, or amiRNA precursor energy variation and modification in the development of the precursor by DCL1 in producing protection from TYLCV infection and the manifestation of virus symptoms. A similar variation in symptoms was also found when transgenic tobacco plants were challenged with CMV (Qu et al., 2007). This variation in transgene expression is affected by the position of insertion, number of transgene copies, and presence of sequences that block transgene expression (Butaye et al., 2005; Sharma et al., 2009). In earlier reports, it was suggested that multiple insertion of the transgene in the plant genome can lead to silencing of the transgene (Shu et al., 2007). In addition, integration into regions of the plant genome that are transcriptionally silent leads to silencing of the transgene. In this situation, transgenic lines with a single insertion could be selected and used (Carter et al., 2002; Gelvin, 2003; Wilson et al., 2006). Moreover, silencing of the transgene could be a result of viral suppressor proteins that inactivate the RNA-silencing mechanism and lead to a break of the resistance (Mansoor et al., 2006; Hohn and Vazquez, 2011). Furthermore, through TEM, aggregates of virus-like particles and the absence of a nucleolus in the parenchymal cells of phloem from inoculated control plants were observed. In contrast, in the nucleolus, no virus-like aggregates were visible in the transgenic samples. Russo et al. (1980) reported the aggregates of virus-like particles in the parenchymal cell of infected tomato phloem. Such particles of 18-20 nm were detected in the control plants, showing symptoms of African cassava mosaic (ACMV) virus using immunosorbent electron microscopy (Frischmuth et al., 2001). Tissues of *N. benthamiana* plants stained with azure A showed aggregates of tomato golden mosaic virus in the nuclei of the phloem and mesophyll cells (Rushing et al., 1987).

In this report, the Rep-amiRNA1 and Rep-amiRNA1 transgenic plants targeting the conserved ends of the Rep gene were shown to confer resistance to TYLCV-OM. Therefore, both ends of the Rep protein of TYLCV are absolutely required for the normal biological functions of TYLCV, particularly in the replication of the virus. It is believed, from this finding, that the v-amiRNA/c-amiRNA mediates the breakdown of one strand of the virus, and subsequently, none of the opposite strand synthesis will occur for TYLCV replication. According to our knowledge, this is the first study of the management of the Rep gene of TYLCV-OM using the amiRNA approach. In future work, our goal is to use amiRNAs that target various other conserved regions to combat the broad spectrum of resistance against different begomoviruses identified in Oman.

## Data availability statement

The original contributions presented in the study are included in the article/**Supplementary Material**. Further inquiries can be directed to the corresponding author.

## Author contributions

MA-R and UA performed the experiment work. JK conceived the idea and supervised the work. AA-S and MS interpreted the data and wrote the manuscript. All authors contributed to the article and approved the submitted version.

## Supplementary material

Supplementary Figure 1 DNA was extracted from two groups of tobacco plants; plants of first group (Lane 1-10) were inoculated with Rep-amiRNA and second group (Lane 11-18) were inoculated with Rep-amiRNA. These plants were used to assess the presence of TYLCV coat protein by PCR. TYLCV CP was detected in symptomatic plants (8, 13, 14) and in symptomless plants (4, 5, 7, 12, 15). A fragment of 650 bp was detected. M= 1kb DNA marker.

Supplementary Figure 2 PCR analysis of F1 tomato plants using 35S promoter. Amplicon of 300bp is observed in all plants (Lane 1-18). P1, P2= Positive control, N= Negative control, M= 1kb marker DNA.

Supplementary Figure 3 PCR analysis of F1 tomato plants challenged with TYLCV using CP-specific primers. Lanes 1-3 are V-amiRNA-transformed plants. Lanes 4-18 are C-amiRNA-transformed plants. Amplicon of 650bp is observed in control plant (Lane 19, 20, 21) and symptomatic transgenic plant (Lane 14). N= Negative control, M= 1kb marker DNA.
