# Supplementary figures and images for: Artificial microRNA-mediated resistance against Oman strain of tomato yellow leaf curl virus
Source: Front Plant Sci. 2023 Mar 30;14:1164921. doi: 10.3389/fpls.2023.1164921 (PMC10098008; doi:10.3389/fpls.2023.1164921)

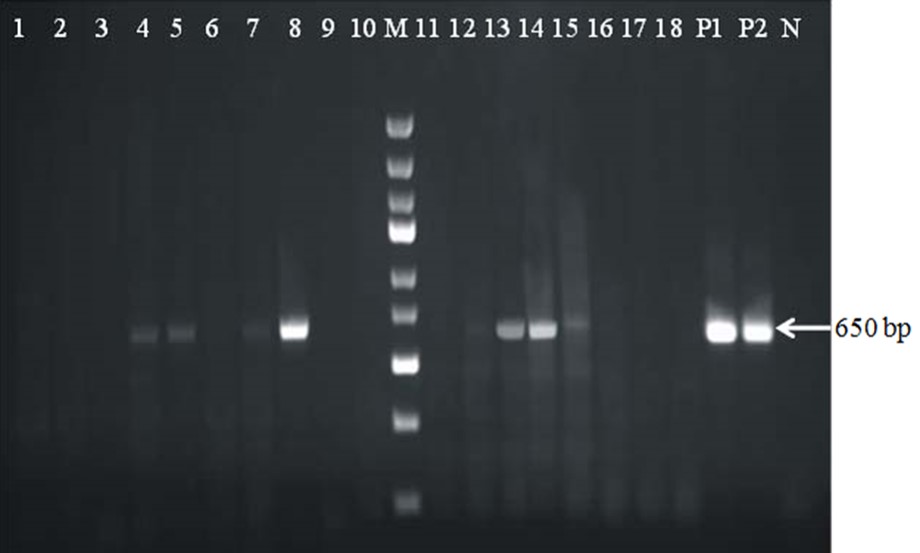

Supplement: Supplementary Figure 1 — DNA was extracted from two groups of tobacco plants; plants of first group (Lane 1-10) were inoculated with Rep-amiRNA and second group (Lane 11-18) were inoculated with Rep-amiRNA. These plants were used to assess the presence of TYLCV coat protein by PCR. TYLCV CP was detected in symptomatic plants (8, 13, 14) and in symptomless plants (4, 5, 7, 12, 15). A fragment of 650 bp was detected. M= 1kb DNA marker. [file Image_1.jpeg]

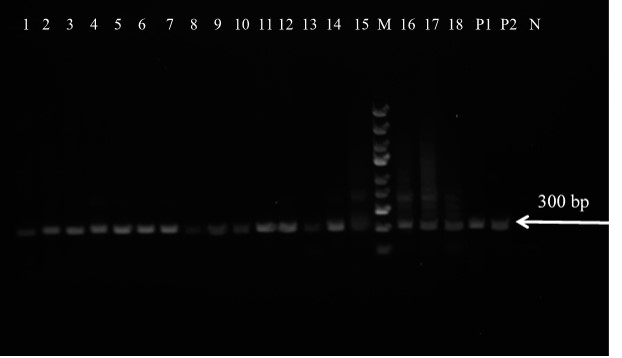

Supplement: Supplementary Figure 2 — PCR analysis of F1 tomato plants using 35S promoter. Amplicon of 300bp is observed in all plants (Lane 1-18). P1, P2= Positive control, N= Negative control, M= 1kb marker DNA. [file Image_2.jpeg]

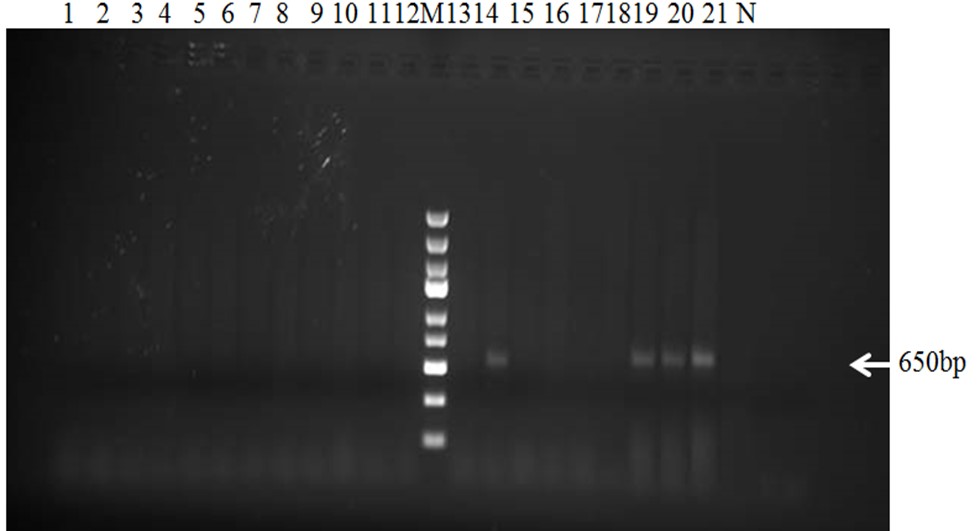

Supplement: Supplementary Figure 3 — PCR analysis of F1 tomato plants challenged with TYLCV using CP-specific primers. Lanes 1-3 are V-amiRNA-transformed plants. Lanes 4-18 are C-amiRNA-transformed plants. Amplicon of 650bp is observed in control plant (Lane 19, 20, 21) and symptomatic transgenic plant (Lane 14). N= Negative control, M= 1kb marker DNA. [file Image_3.jpeg]
